# Autosomal dominant Alzheimer's disease in Argentina

**DOI:** 10.1002/alz70858_103636

**Published:** 2025-12-26

**Authors:** Maria Eugenia Sarandria

**Affiliations:** ^1^ FLENI, Buenos Aires, Ciudad Autonoma de Buenos Aires, Argentina

## Abstract

**Background:**

Genetic factors play a significant role in Alzheimer's disease development, with mutations in genes such as APP, PSEN1, and PSEN2 being linked to familial forms. We aim to describe the clinical, neuroimaging and neurocognitive profiles of Argentine patients with genetic Alzheimer's disease.

**Method:**

This is a cross‐sectional observational study. We described a cohort of Argentine patients diagnosed with genetic Alzheimer's disease. Sociodemographic data of the patients were collected, including education and ocupation. Patients were assessed through neurocognitive evaluations, magnetic resonance imaging (MRI), and positron emission tomography (PET) with PIB and FDG. The participants were selected based on the presence of known genetic mutations (PSEN1, PSEN2, APP) associated with Alzheimer's disease. Neurocognitive assessments included a battery of clinical tests to evaluate cognitive domains such as memory, executive function, attention and language (CDR, Age at onset evaluation, Neurologic exam, etc). MRI scans were used to examine structural brain changes, while PET imaging was employed to assess metabolic activity (FDG) and amyloid deposition (PIB).

**Result:**

We studied a total of 26 total (males: 12). The majority (80%) presented a mutation in PSEN1. At baseline evaluation, the cohort exhibited distinct neurocognitive impairments, primarily affecting memory and executive functions. MRI findings revealed significant cortical atrophy, particularly in the hippocampal and parietal regions, while PET scans showed elevated amyloid deposition and reduced glucose metabolism in areas associated with Alzheimer's pathology.

**Conclusion:**

Our study provides valuable insights into the clinical and neuroimaging characteristics of Argentine patients with genetic Alzheimer's disease. The combination of neurocognitive assessments, MRI, and PET imaging offers a comprehensive approach to understanding the disease's progression and may inform future diagnostic and therapeutic strategies.

